# The Views of Hospital Secretaries

**Published:** 1911-05-20

**Authors:** 


### THE VIEWS OF HOSPITAL SECRETARIES.

**Mr. LEONARD D. REA, Secretary of Cardiff Infirmary, writes:—**

The hospitals cannot be disregarded in any national scheme of insurance against sickness, and it is apparently assumed that they will be continued under present conditions, for no provision has been included in the Bill for their maintenance, as in the case of consumption sanatoria, and the bulk of hospital work (including, as it does, major surgical operations, requiring an infinity of detail of a highly technical nature) lies entirely outside the scope of the proposed legislation.

The efficiency and the maintenance of the Cardiff Infirmary at present depend upon the honorary medical staff, the general subscribers, the colliers, and Cardiff and district workmen not associated with the colliery industry. Beyond expressing a sincere acknowledgment of the splendid services so freely and so ungrudgingly given by the medical profession, and which form the keystone of the voluntary edifice, I would not venture an opinion as to any probable modification in this respect, induced by the operations of the proposed Bill. That is a matter primarily for the medical profession. There may be a temptation for the general subscriber to withhold his subscription if the insurance scheme makes a large demand upon his resources, as an employer, but I feel confident that every fair-minded man will continue his support of an efficient hospital while the necessity exists, and this not only from philanthropic reasons but in recognition of its importance, from an economic point of view, in maintaining the validity of the community.

As to the continuance of contributions by the colliers, I have no shadow of doubt. The Welsh collier may be slow in giving his confidence, but he is equally slow in withdrawing it. He regards the Infirmary with affection, and I believe that his levy for the Infirmary would be one of the last to be disregarded. It should be borne in mind that he contributes to a variety of objects, so the individual contribution to the Infirmary is nominal, but the aggregate of colliers' contributions realises a handsome amount. As to the workman who contributes at present a penny a week, a hospital secretary must above all things be optimistic, and as a second line of defence one may refer with gratitude to Clause 17 of the Bill, which permits an approved Friendly Society to grant subscriptions to hospitals, but one may be permitted to express the hope that such subscriptions may not be required to make up for any diminution in the voluntary contributions of working people. It is in one sense idle to speculate upon the future of a hospital, but it is of the utmost importance—now more so than ever—to maintain the highest possible standard of service and efficiency, and to assure by intelligent appeal and unwearying effort that the actual existence of the hospital is kept constantly before all sections of the community. As in other spheres, the best defence of a hospital lies in the power and the means of instructing the public in the nature and scope of its work and its consequent requirements. Should, in the process of evolution, a partial or complete contribution be afforded to such institutions by the State, it is earnestly to be hoped opportunity will be left open for utilising the services of voluntary workers, to whom the hospitals of the kingdom at present owe all that is best in their development. I do not think that any system, confined by necessity within official lines, could with so great a measure of success and with such an enlightened regard to economy have advanced the Cardiff Infirmary to its present position more than the unselfish and devoted but also far-seeing and practical efforts of the Chairman of our House Committee, Col. Bruce Vaughan.

It is significant that the collier, whose ideas are usually progressive, has never suggested the assumption of hospital control by the State or the municipality. With others, unacquainted with the Infirmary and usually uninformed on the subject, I can always come to agreement in the proposition that, in any case, pending the slow process of legislation, the hospitals must continue to be maintained. It is a homely argument "If your child falls into the river, must you wait for the policeman to fetch him out, or may not you or I attempt a rescue? The policeman may arrive too late."

**Mr. FRED J. BRAY, General Manager of the General Infirmary, Leeds, writes:—**

The Insurance Bill will, I think, have the effect of reducing the attendance in our out-patient department, and then it will obtain its legitimate object in being a "consultation department"; this has been our aim for some time. The demand on our beds will doubtless be increased, as numbers who now make an effort to nurse their sick at home, unwilling to accept charity, will consider themselves entitled to hospital treatment at the expense of the Insurance Fund. How these changes will influence our income is a moot point.

The workpeople's Hospital Fund will probably become extinct—at any rate, that portion now given to medical charities. This probably will be more than compensated for by the subscriptions from the Medical Committee or Society, nearly the whole, if not all, our patients coming within the compulsory clauses. The necessary income for the upkeep of our contemplated extensions will only be obtained with considerable difficulty, but I anticipate not more so under the changed conditions. Whilst I am in full sympathy with the object of this legislation, I fear it is only the beginning of all our hospitals becoming municipalised.

**Mr. W. THWAITES, Secretary of the Bristol General Hospital, writes:—**

The effect of the new Insurance Bill on this hospital is likely, I believe, to be a very large reduction in the number of out-patients treated and a corresponding reduction in the contributions received from the working classes. These as a body give with the object of receiving hospital admission notes when needed. Probably the opportunity for teaching in both the medical and nursing professions will be much reduced, and this must be a heavy loss to hospitals and the public generally.

**Mr. E. C. SMITH, Secretary of the North Ormesby Hospital, Middlesbrough, writes:—**

I consider that the hospital relying mostly upon annual subscriptions will suffer financially most, as practically every annual subscriber is an employer to a certain extent, and when in future he is called upon to pay insurance for each of his clerks and domestic servants he may probably reduce or stop his subscriptions to hospitals, thinking he has done quite enough for the class of persons using hospitals in providing them with sick pay, more especially so as he will probably have to pay the 2d. provided by the State in increased taxation.

As four-fifths of the income of the hospital of which I am Secretary are provided by workpeople, and the greater portion of the remaining one-fifth by employers, I have considered this question very carefully. In proportion to his income the workman is a most generous giver (particularly in the North). He pays 7d. to his Friendly Society, 3d. or 4d. to his doctor, and 1d. or 2d. to the hospital each week; in addition he gives to the Hospital Saturday and Sunday Funds, Dr. Barnardo's Homes, Nursing Institution, and to some other special objects. It will be a great strain for him to give another 4d. or 6½d. weekly, so he will probably reduce his present subscriptions. He clearly recognises that hospitals are most necessary to him and must be maintained, so he will not reduce his subscription to them. The Bill provides him with "free doctors and medicine," but apparently makes no provision for his wife and children, so he may continue to subscribe to his club doctor. He will be entitled to the same amount in sick pay as he receives at present from his Friendly Society, so he will most probably reduce his subscriptions to Friendly Societies, more especially so as he is sometimes a member of more than one society.

Large employers of labour have informed me that they consider their subscriptions to hospitals as a necessary expenditure, not a gift, because they say hospitals assist in making a man fit for work again in the minimum time, and so save the employers many hundreds of pounds in compensation; consequently they will not stop their subscriptions, but the person who only employs two or three people may stop or reduce his subscription.

On the whole, I do not think hospitals in industrial centres will be affected financially, apart from what they will pay on their staffs, to any appreciable extent, but the number of cases applying for admission will probably show a big increase, and malingerers—consisting chiefly of unemployables—will assuredly be very much in evidence.

